# Processing of boar spermatozoa with phosphate-buffered saline at 4˚C induces an increase in 32 kDa tyrosine-phosphorylated protein (p32)

**DOI:** 10.1007/s11259-023-10247-2

**Published:** 2023-10-27

**Authors:** David Martín-Hidalgo, Mercedes Izquierdo, Paloma Bartolomé-García, Beatriz Macías-García, Lauro González-Fernández

**Affiliations:** 1 Departamento de Bioquímica y Biología Molecular y Genética, Grupo de investigación Señalización Intracelular y Tecnología de la Reproducción (SINTREP), Facultad de Veterinaria, Instituto de Investigación INBIO G+C, Universidad de Extremadura, Av. de las Ciencias, s/n, Cáceres, 10004 Spain; 2 Centro de Investigaciones Científicas y Tecnológicas de Extremadura (CICYTEX), Badajoz, Spain; 3 Centro de Selección y Reproducción Animal (CENSYRA), Badajoz, Spain; 4 Departamento de Medicina Animal, Grupo de investigación Medicina Interna Veterinaria (MINVET), Instituto de Investigación INBIO G+C, Facultad de Veterinaria, Universidad de Extremadura, Cáceres, Spain

**Keywords:** Boar, Spermatozoa, Capacitation, p32

## Abstract

We aimed to investigate the impact of processing boar spermatozoa with phosphate-buffered saline (PBS) at 4 ˚C on acrosomal integrity and increase in 32 kDa tyrosine-phosphorylated protein (p32). Following cooled PBS washing, we observed a significant increase in p32 levels and in the proportion of dead spermatozoa with compromised acrosomal integrity compared to sperm washing using PBS at room temperature. Interestingly, this increase in p32 was effectively inhibited when cooled PBS was supplemented with 1 mM AEBSF, a serine protease inhibitor. Our findings suggest that the increase of p32 in response to cooled PBS washing in boar spermatozoa is associated with enhanced protease activity in dead spermatozoa.

## Introduction

Capacitation is a complex phenomenon that occurs in the female reproductive tract and involves numerous changes in spermatozoa, which are required to attain fertilization of the oocyte (Austin 1951; Chang 1951). One of most studied hallmarks of capacitation is the increase in protein phosphorylation of tyrosine residues (protein tyrosine phosphorylation or PY) (Visconti et al. 1995). In boar spermatozoa, the 32 kDa tyrosine-phosphorylated protein (p32) is used as a marker for capacitation (Tardif et al. 2001). The importance of the study of p32 lies in its unique behaviour during capacitation in boar spermatozoa. Unlike many other species, p32 is the only protein consistently undergoing modifications under capacitation conditions, without the need for the addition of external agents to induce this phenomenon. It is known that the increase of p32 under capacitating conditions is dependent of extracellular calcium (Dube et al. 2003) and has been associated with an increase in the percentage of acrosome-reacted spermatozoa (Harayama et al. 2004; Martin-Hidalgo et al. 2022). However, p32 can also increase after exposition to sperm-stressing conditions, such as cold shock (10 min at 10 ˚C) (Galantino-Homer et al. 2006). In this regard, Tabuchi et al. observed a correlation between p32 increase and acrosomal damage in boar spermatozoa after rapid thawing at 38.5 ˚C, suggesting an association between p32 increase and proteolysis (Tabuchi et al. 2008).

To assess p32, different techniques can be used, with western blotting being the most commonly used. In western blotting, cells must be lysed to obtain the protein extracts that will be analysed. Prior lysis, to minimize non-specific interferences, cells are typically washed to remove the incubation medium. Generally, cell lysis protocols include a washing step of the cells with phosphate-buffered saline (PBS), which is recommended to be maintained at 4 ˚C. This low temperature is essential to protect proteins from the activities of proteases or phosphatases that could alter the results (Litovchick 2018; Mahmood and Yang 2012). However, it is crucial to note that spermatozoa are highly sensitive to temperature changes, and cold shock can induce sperm damage due to lipid phase transitions in the cell membranes (Drobnis et al. 1993). This cold-induced damage can result in acrosomal and plasma membrane damage (Darin-Bennett et al. 1973; De Leeuw et al. 1990), contributing to the release of proteases from the acrosome. Therefore, the objective of this study was to analyse the influence of cooled PBS (at 4 ˚C) used for sperm washing before lysis on p32 and acrosome integrity in boar spermatozoa.

## Materials and methods

### Media

Tyrode’s complete medium (TCM) consisting of 96 mM NaCl, 4.7 mM KCl, 0.4 mM MgSO_4_, 0.3 mM NaH_2_PO_4_, 5.5 mM glucose, 1 mM sodium pyruvate, 21.6 mM sodium lactate, 20 mM HEPES, 1 mM CaCl_2_, 15 mM NaHCO_3_ and 3 mg/ml BSA. Bicarbonate was added 1 h prior to the experiment and adjusted to a pH of 7.45. Phosphate-buffered saline (PBS) was composed by 2.7 mM KCl, 1.75 KH_2_PO_4_, 136.9 mM NaCl, 8 mM Na_2_HPO_4_ and adjusted to a pH of 7.45. All reagents were purchased from Sigma-Aldrich (St. Louis, MO, USA), unless otherwise stated.

### Semen collection and processing

Seminal doses were purchased from a commercial boar station (Tecnogenext, S.L., Mérida, Spain). Duroc boars were maintained according to Regional Government and European regulations. For this study, there was no handling of the animals; therefore, the authorization of an ethics committee was not considered necessary. Three seminal doses were mixed from three different males per experiment, centrifuged at 900 × g for 4 min at room temperature (RT; 22–25˚C) and washed with PBS at RT. Spermatozoa were diluted in TCM and adjusted to a final concentration of 50 mill/ml and divided in five groups (500 µl final volume). Aliquots of the final sperm suspension were placed in 5 ml round bottom plastic tubes from Falcon (Corning, NY, USA) and incubated for 2 h in a water bath at 38.5 ˚C in air. After incubation, spermatozoa were centrifugated (3 min at 5000 × g) at RT and each sample was washed in PBS at RT or in cooled PBS (at 4 ˚C). The washing procedure consisted on the resuspension of the cells in 1 ml of PBS. To investigate the involvement of extracellular calcium and serine proteases on the effects of cooled PBS washing, cooled PBS was supplemented with ethylene-bis (oxyethylenenitrilo) tetraacetic acid (EGTA) at 5 mM or the general serine protease inhibitor 4-(2-aminoethyl) benzenesulfonyl fluoride hydrochloride (AEBSF) at 0.1 or 1 mM. After PBS washing, spermatozoa were analysed by flow cytometry or lysed for p32 analysis using western blotting.

### Western blotting

After centrifugation (3 min at 5000 × g at RT), spermatozoa were processed as described previously by González-Fernández et al. (González-Fernández et al. 2013). Briefly, spermatozoa were lysed in Laemmli buffer 2X (Bio-Rad, Hercules, CA, USA) and the protein content was determined using the Bio-Rad DC Protein Assay (Bio-Rad, Hercules, CA, USA). Samples (15 µg of protein) were loaded and electrophoresed in 10% SDS-polyacrylamide gels and transferred to PVDF membranes (Merck KGaA, Darmstadt, Germany). Membranes were incubated overnight at 4 ˚C with an anti-phosphotyrosine monoclonal antibody (clone 4G10) (#05-321; Merck KGaA, Darmstadt, Germany) diluted 1:5000 (v/v) in Tris-buffered saline-Tween 20 solution (TBS-T) containing 3% of BSA. α-Tubulin levels were used as a loading control; membranes were incubated with an anti-α-tubulin antibody (#sc-8035; Santa Cruz Biotechnology, CA, USA) diluted 1:5000 (v/v) in TBS-T with 3% BSA overnight at 4 ˚C. Then, membranes were incubated for 45 min at RT with a secondary anti-mouse antibody conjugated to horseradish peroxidase (#sc-516,102; Santa Cruz Biotechnology, CA, USA) diluted 1:5000 (v/v) in TBS-T containing 3% BSA. Following secondary antibody incubation, membranes were washed for 20 min in TBS-T, then incubated with Supersignal™ West Chemiluminescent Substrate (Thermo Fisher Scientific Inc., Waltham, MA, USA) and exposed to Hyperfilm™ ECL (Amersham, Arlington Heights, IL, USA). Densitometric analysis was performed using Gel-Pro Analyzer™ ver. 4.0 (Media Cybemetics, Bethesda, MD, USA). This experiment was performed 5 times (n = 5).

### Flow cytometry

Samples were analysed 3 min after performing the washing procedure using an ACEA NovoCyte™ flow cytometer (ACEA Biosciences, Inc., San Diego, CA, USA) equipped with a blue/red laser (488/640 nm). ACEA Novo Express™ software (ACEA Biosciences, Inc., San Diego, CA, USA) was used for experiments and data analysis. Forward scatter (FSC) and side scatter (SSC) were used to gate the sperm population and exclude debris. Spermatozoa were analysed at a rate of 400–800 cells/s, and data were collected for 10,000 cells in each treatment. Before flow cytometry analysis samples were diluted at 1–3 mill/ml in 500 µl of PBS at RT. Acrosome integrity and viability were assessed using peanut agglutinin conjugated to fluorescein isothiocyanate (PNA-FITC) and propidium iodide (PI) (Martin-Hidalgo et al. 2022). PNA-FITC and PI were added to samples at 0.4 µg/ml for PNA-FITC and at 4.8 µM for PI. After 5 min in the dark at RT, PNA-FITC and PI fluorescence were detected using 530 ± 30 nm and 675 ± 30 nm band pass filters, respectively. Acrosome-damaged spermatozoa are expressed as the percentage of PNA-positive cells (PNA^+^). This experiment was performed 5 times (n = 5).

### Statistical analysis

Analyses were performed using SigmaPlot software (ver. 12.0) for Windows (Systat Software, Chicago, IL, USA). Percentage data from flow cytometry experiments were arcsine-transformed before statistical analysis. All data are shown as the mean ± standard error of the mean (SEM). An ANOVA followed by Dunnett’s post-hoc test was used to compare different treatments to control (PBS at RT). The level of significance was set at P < 0.05.

## Results

**Fig. 1:**
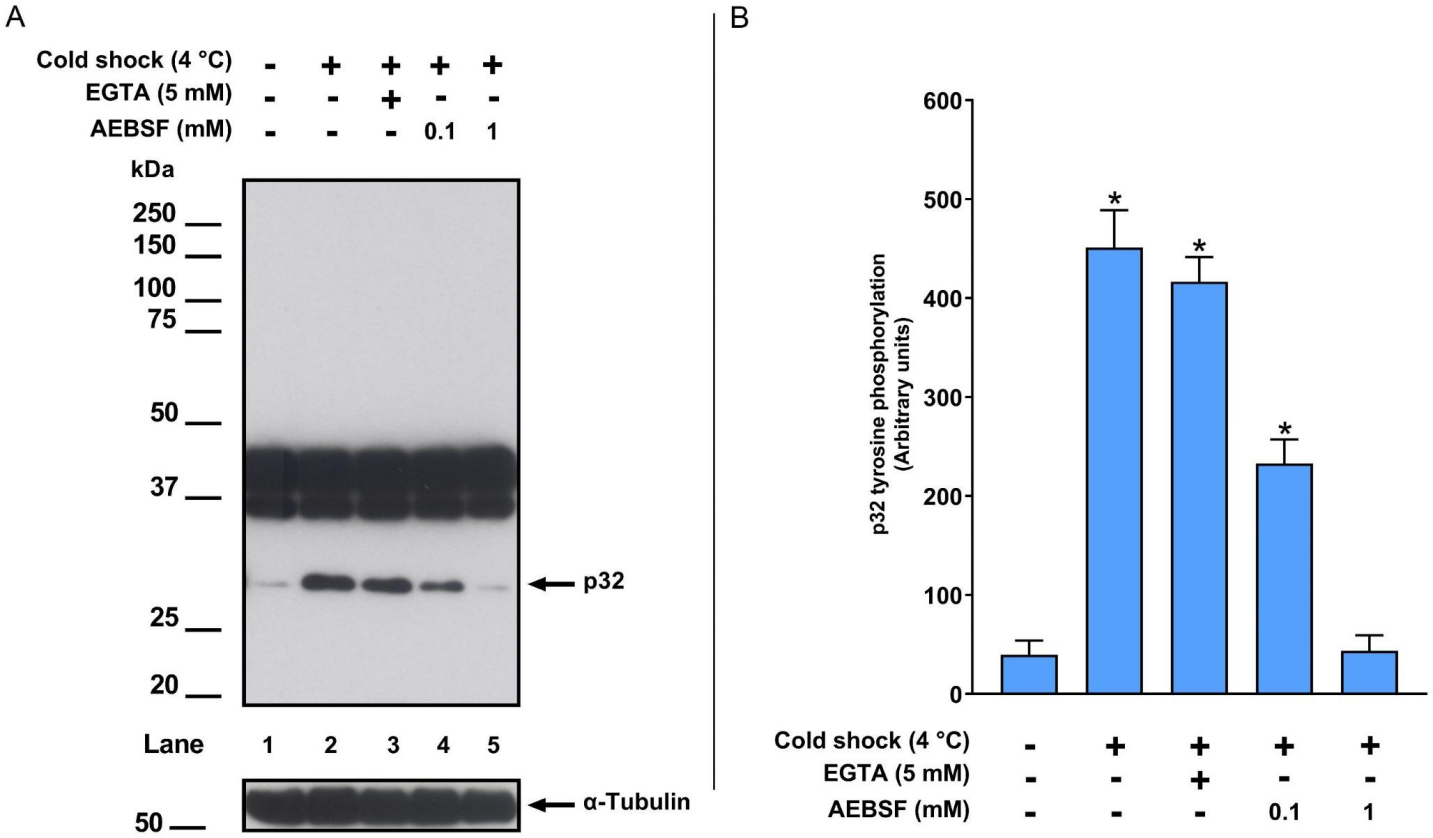
Effect of PBS temperature on protein tyrosine phosphorylation. Boar spermatozoa were incubated in TCM for 2 h at 38.5 ˚C. After incubation, spermatozoa were washed in PBS at room temperature (control) or at 4 ˚C before cell lysis. (A) Western blotting was performed using an anti-phosphotyrosine antibody (n = 5). α-Tubulin was used as loading control. (B) The bars represent the mean ± SEM of the optical densities of p32. Bars marked with an asterisk differ at P < 0.05 from the control

The results demonstrated a significant increase in p32 levels when boar spermatozoa were washed with cooled PBS compared to PBS at RT (Fig. 1). The presence of EGTA in cooled PBS did not inhibit this increase in p32 levels (Fig. 1). However, when cooled PBS was supplemented with 1 mM AEBSF, a significant decrease in p32 levels was observed (451.13 ± 37.91 [cooled PBS] vs. 43.77 ± 15.68 [cooled PBS + 1 mM AEBSF], mean arbitrary units ± SEM, P < 0.05; Fig. 1B).

**Fig. 2:**
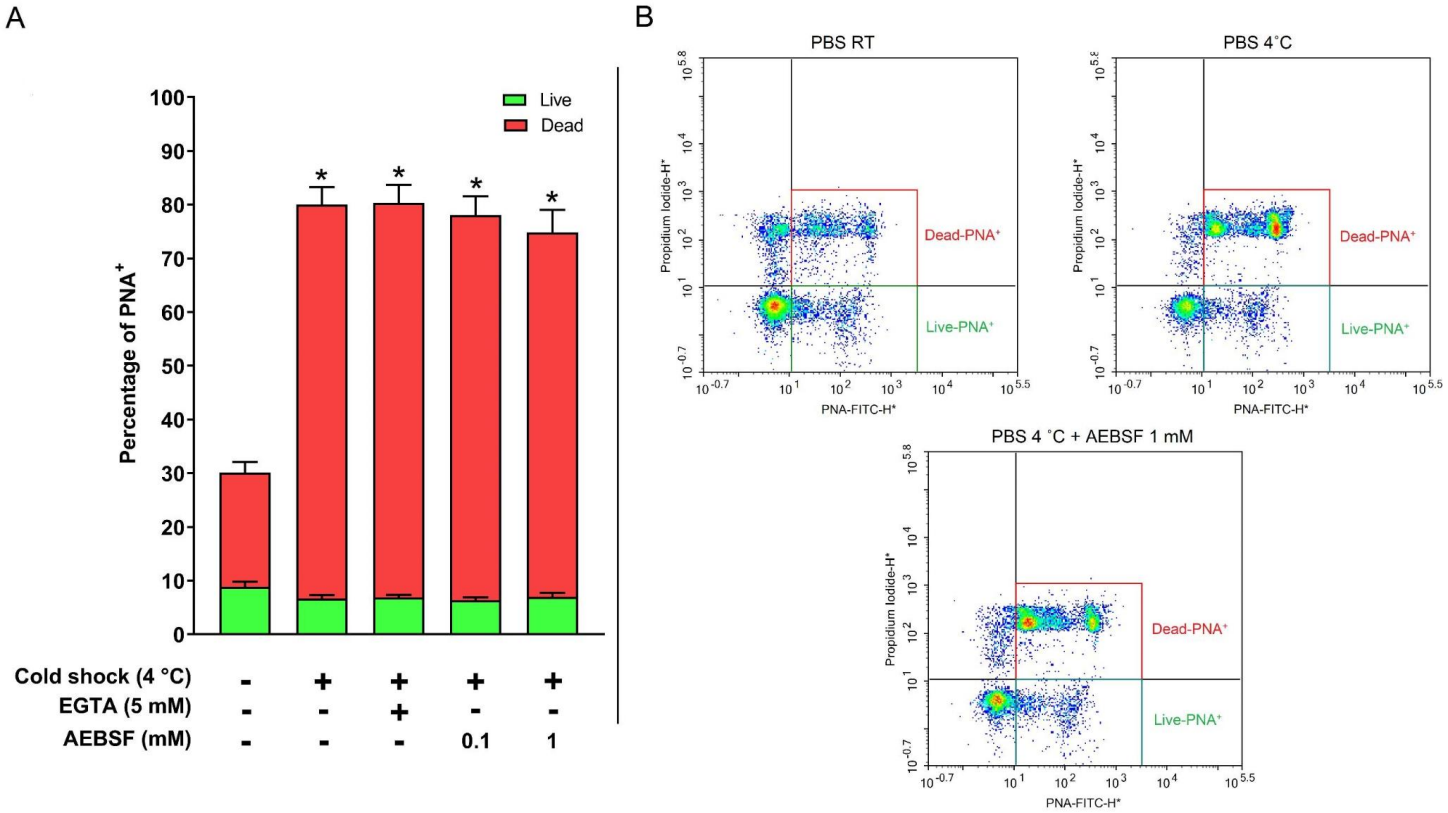
Effect of PBS temperature on acrosome integrity and viability. Boar spermatozoa were incubated in TCM for 2 h at 38.5 ˚C. After incubation, spermatozoa were washed in PBS at room temperature (control) or at 4 ˚C and acrosome integrity and viability were simultaneously assessed by flow cytometry using PNA-FITC and PI. (A) The percentage (mean ± SEM) of live or dead spermatozoa with damaged acrosome (PNA^+^) are shown in green and in red, respectively (n = 5). Bars marked with an asterisk differ at P < 0.05 from the control. (B) Representative dot plots for the different treatments

When we analysed the effect of cooled PBS on acrosome status, we observed a higher percentage of dead spermatozoa with acrosomal integrity loss (Fig. 2) compared to the control (PBS at RT), and the presence of EGTA in cooled PBS had not effect. The percentage of dead spermatozoa with acrosomal integrity loss was not significantly affected by the addition of 1 mM AEBSF (73.35 ± 3.27 [cooled PBS] and 67.91 ± 4.20 [cooled PBS + 1 mM AEBSF], mean % ± SEM, P > 0.05; Fig. 2A).

## Discussion

Our results demonstrate that washing spermatozoa with PBS at 4 ˚C leads to acrosomal damage and a significant increase in p32 levels, which could be erroneously associated with sperm capacitation. We showed that washing spermatozoa with cooled PBS containing 1 mM AEBSF prior to cell lysis effectively inhibited the increase in p32 levels (Fig. 1), suggesting the implication of acrosomal proteases as approximately 80% of spermatozoa underwent acrosomal damage (Fig. 2). Our findings are consistent with those of Tabuchi et al. (2008), who described a decrease in p32 levels in boar spermatozoa subjected to rapid thawing in PBS using APMSF, another serine protease inhibitor. Furthermore, we observed that the presence of EGTA in the PBS did not inhibit p32 increase. Our result is in accordance with Tabuchi et al. (2008), suggesting a different mechanism for p32 increase in dead spermatozoa, as extracellular calcium was not necessary for p32 rise. These authors proposed that p32 increase could be linked to the release of acrosin from the acrosome which is a protein with serine protease activity. Our laboratory recently demonstrated a novel mechanism for p32 rise involving the proteolysis of glycosylated form of SPACA1 in boar spermatozoa (Macías-García and González-Fernández 2023). We proposed that SPACA1 (35–45 kDa), a transmembrane protein located in the inner acrosomal membrane, is exposed upon acrosome loss, and is subsequently cleaved by proteases to generate the 32 kDa form of SPACA1 (p32). Altogether, our results demonstrate that when the acrosome is damaged due to cold stress, proteases contained in the acrosome can still exert their function at low temperature, inducing an increase in p32 levels unrelated with true capacitation. The specific identity of the proteases involved in p32 increase under these stressing conditions remains unknown and requires further investigation.

## Conclusion

In conclusion, our study emphasizes the importance of utilizing washing media at room temperature before the sperm lysis step to avoid acrosomal damage that may interfere with p32 analysis. Our findings should be considered when studying other proteins in different mammalian species using alternative techniques to ensure accurate results and avoid potential artifacts that could lead to erroneous conclusions.

## Data Availability

The data that support the findings of this study are available on request from the corresponding author.
